# Models for SARS-CoV-2 health policies: social distancing amid vaccination and virus variants

**DOI:** 10.1098/rsos.242277

**Published:** 2025-10-22

**Authors:** Yi Zhang, Sanjiv Kapoor

**Affiliations:** ^1^ Illinois Institute of Technology, Chicago, IL, USA; ^2^ Department of Computer Science, Illinois Institute of Technology, Chicago, IL, USA

**Keywords:** models, SARS, CoV-2, health, policies, epidemiological models

## Abstract

Policy decisions during the SARS-CoV-2 pandemic were important to contain the spread of the virus, but complicated due to virus variants and the varying impact of societal restrictions. In this paper, we report results from a model that utilized population behaviour to predict the impact of SARS-CoV-2 transmission in India over a number of months from June 2021 to March 2022. The model utilizes deterministic population compartments, incorporating a dynamic transmission factor dependent on the population’s behaviour as a function of reported confirmed cases. The model also incorporates the state of vaccination and virus variants as part of the transmission dynamics. The model projections, used for formulating advice towards pre-emptive policy actions by pivotal government organizations involved in developing a national public health strategy, culminated in early warning projections for the Omicron variant. The projections of cumulative cases during the Omicron wave had a mean absolute percentage error of 18.1% when measured 15 days after the start of the projection on 1 December 2021.

## 1. Introduction

The SARS-CoV-2 virus has been infecting human populations across the world since January 2020, compounding the original problem with multiple variants. Containment measures such as social distancing are the key, even in the presence of vaccinations, to preventing the spread of the virus and reducing the risk of new variants. Models that predict the growth in infections are very important and while short-term analysis of a few weeks is typically more accurate, long-range models are important to inform policy and planning. In this paper, we consider a model for long-term virus infection spread that accounts for the impact of lockdown policies, vaccinations and virus variants.

Effective public health policy requires timely social distancing guidelines; while achieving long-term accuracy is extremely challenging, it is critical to develop early-warning systems that would predict the rise of the virus, especially as physical distancing and other non-pharmaceutical intervention (NPI) policies are relaxed. These warning systems would include parameters that reflect the relaxation of NPI practices related to masking, enhanced mobility and so on, and would be indicative of behaviour patterns that create an influx of susceptible into the population. Additionally, since the spread of the virus in asymptomatic population and even in the vaccinated population results in prolonged impact on the progression of the disease [1], long-term modelling of the spread of the virus is critical.

This paper deals with modelling and analysis of the spread of infection as populations emerge from lockdown and adopt lax behaviour with respect to social distancing. The impact of lax NPI norms on the increase of the daily growth of cases has already been illustrated in [2], while other studies found that social distancing policies decrease COVID-19 growth rates [3,4]. Here, we construct a predictive model that reflects changes in the lockdown policy while accounting for vaccination status of the population, the time-dependent efficacy of the vaccines and the impact of virus variants. The analysis is based on epidemic spread models that utilize compartment models and have a long history (see [5–9]).

The SIR model and its variants represent a class of ‘parsimonious’ models that along with individual-based simulation models [10] could be used to inform pandemic-related policies [11]. While compartment models have been used to model the COVID-19 pandemic extensively in the scientific literature, we mention here two examples where compartment models have been used to provide policy advice in US states, one in Illinois [12] and the other in Texas [13]. A version of the SIR model [14] has also been used to model the pandemic in India as part of governmental policy in the first phase and also subsequently. Furthermore, variation of the SIR model that incorporates presymptomatic and asymptotic populations have been used to study the growth of the virus in Italy [15]. In [16], a semi-time SIR model is used, assuming a constant ratio between infection and recovery rates. The semi-time SIR model can be used with any initial condition, makes predictions only about the future and was used to model a scenario with multiple waves. The constant ratio between infection and recovery rates appears to be a limitation. Typically, the SIR model assumes homogeneity in the contact patterns which does not consider non-homogeneous populations as discerned by ‘disordered’ spatial patterns of infectious cases. To account for this, research by Geng *et al*. [17] uses a modified SIR with a kernel-modulated approach to characterize the dynamics of the disease spread.

Our model has multiple distinct features.

(i) A key differentiation in our model, termed the SIR-SD model, is the use of a dynamic virus transmission rate as introduced by us in an earlier work [18]. This function, which incorporates a factor that is inversely dependent on the number of cumulative infections, reflects the behaviour of the population as the number of infection cases rise. The role of reducing contacts to reduce transmission is well studied [19–21] and more recent modelling efforts have focused also on mobility [22] and time variations of SIR parameters [14], among other factors [23–31].
(ii) Additionally, we incorporate compartments that reflect the various states of the population, including the vaccinated population and a population under lockdown. The lockdown population compartment has been used earlier in our prior work in modelling the growth of the pandemic when NPIs were removed [18]. In other approaches, the model in [13] modifies the transmission rate for the entire susceptible population to account for lockdown, while the model in [32] modifies the susceptible population to account for surges. The lockdown compartment used in this paper is non-standard; we incorporated the additional special state of the population to distinguish it from a section of the population that was not under lockdown. An advantage of such a compartment is that, when government restrictions were relaxed, the population of the compartment representing lockdown returned to the susceptible compartment and its impact on the increase of infections could be studied. The impact of increase in susceptibles is a major factor in the spread of infections [33].
(iii) We incorporate the impact of virus variants in our model. This was used to provide estimates of the impact of the emerging Omicron variant while the Delta variant was still active. Other mathematical analysis of transition from one variant to another may be found in [34], which predicts that dominant strains evade immunity. Most research including the one in [35] assumes cross-immunity.

This work was motivated by the problem of determining policy decisions as vaccinations progressed and we used this model to provide monthly updates on projections to a pivotal Indian government organization, NITI Aayog. The projections focused on the onset of the third wave and covered the period from June 2021 till March 2022. The third wave in India started in December 2021. The second wave had come as a surprise to policy makers and overwhelmed the medical system, as well as resulting in a large number of deaths. Discussions with NITI Aayog led to consideration of multiple aspects including vaccination rates and, as per personal communication, the projections in this paper as detailed in §3.2. were considered when formulating national policy advice. The policies implemented at state level included mask mandates, partial curfews and closure of schools and offices, as illustrated by Delhi state government policies [36].

## 2. SEIAR-SD-L model

**Figure 1.**
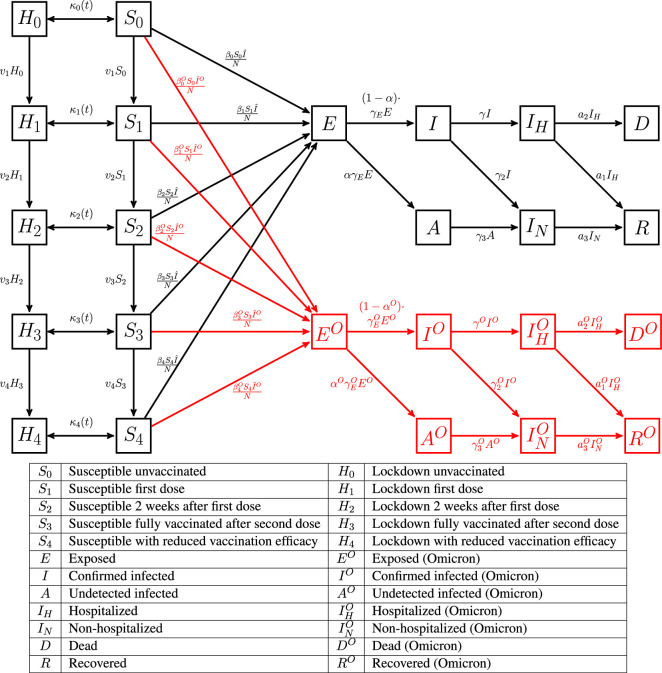
The SEIAR-SD-L compartment model with vaccination and compartments for two strains of the virus: Delta and Omicron (red). The compartments $S_{0}\ldots S_{4}$ represent the susceptible population in various stages of vaccination. $H_{0}\ldots H_{4}$ represent the hidden populations that has been isolated. Asymptomatic and undetected infectious population is in the group A (infected by the Delta variant) and $A^{0}$ (infected by the Omicron variant).

We use a mechanistic model as illustrated in figure 1 and table 1. Apart from standard compartments representing the susceptible S, exposed E, infected (confirmed via testing) I, undetected symptomatic and asymptomatic A, we include hospitalized *I*_H_ and home-treated *I*_N_, along with dead D and recovered R. For simplicity, we assume that anyone who dies due to the virus is on the path from I to *I*_H_ to D; this accounts for COVID-19 deaths as reported. We do not have hospitalization data so this path is not constrained during model fitting. If hospitalization data were available then the compartments would be modified to account for recognized deaths among home-treated and asymptomatic/undetected populations. Additionally, we include compartments representing the population under lockdown which are discussed next.

**Table 1.** Table of parameters for the SEIAR-SD-L model. Compartments and transitions corresponding to the Omicron variant are indexed by O.

$\kappa_{i}(t)$	transmission between lockdown and susceptible compartments	* $v_{i}$ *	transition rate between vaccinated states
$\beta_{i}^{O}$	Viral transmission rate between *S*_*i*_ and *E*	$\beta_{i}$	viral transmission rate between $S_{i}^{O}$ and *E*
* $\gamma_{E}$ *	transition rate between exposed and infected sets	α	fraction of people infected asymptomatically
$\gamma_{E}^{O}$	transition rate between *E*^O^ and *I*^O^ for Omicron variant	$\alpha^{O}$	fraction of people infected asymptomatically with Omicron variant
* γ *	transition rate from infected to hospitalized	* $\gamma_{2},\gamma_{3}$ *	transition rate from infected to non-hospitalized
* $\gamma^{O}$ *	transition rate from infected to hospitalized for Omicron variant	$\gamma_{2}^{O},\gamma_{3}^{O}$	transition rate from infected to non-hospitalized for Omicron variant
* $\alpha_{2}$ *	transition rate from hospitalized to death	* $\alpha_{1},\alpha_{3}$ *	transition rate from hospitalized to recovered
$\alpha_{2}^{O}$	transition rate from hospitalized to death for Omicron variant	$\alpha_{1}^{O},\alpha_{3}^{O}$	transition rate from hospitalized to recovered for Omicron variant

The lockdown and susceptible compartments are grouped by different vaccination states, as follows. $H_{0}$: the set of unvaccinated population under lockdown. $H_{1}$: the population that has received the first shot of vaccination (based on the rate of vaccination of the population). $H_{2}$: population after two weeks of receiving the first dose (first dose effective two weeks after the dose). $H_{3}$: population after two weeks of receiving the second dose of vaccination given after a fixed delay from the first dose. $H_{4}$: vaccinated population after 4 months of the second dose. Similarly the vaccination status of the susceptible population is modelled by $S_{0}$, $S_{1}$, $S_{2}$, $S_{3}$ and $S_{4}$. Each such compartment is characterized by reduction in virus transmission rate (from the susceptible compartments to the exposed compartments) via a parameter termed as the vaccine efficacy ζ. We assume that after 2 weeks of the first dose, the vaccine efficacy (reflected by reduction in $\beta_{2}$ for $S_{2}$) is $\zeta_{2}$. After being fully vaccinated, the vaccine efficacy is represented by $\zeta_{3}$. Vaccination efficacy has been known to drop by 50% after 4 months and $H_{4}$ and $S_{4}$ model the population in this state [37], and thus $\zeta_{4}$, the vaccine efficacy of the population in $S_{4}$ is reduced to $\frac{\zeta_{3}}{2}$. We will let H and S represent the population in all the lockdown and susceptible compartments together, respectively.

In the model, in addition to the currently prevalent Delta variant of the virus we used compartments for the Omicron virus variant. The disease specific compartments are replicated for each virus variant to model the individual impact of each variant. The replicated compartments are E, I, A, $I_{H}$, $I_{N}$, D and R, and these compartments are indexed by superscript *O*. All the compartments and transitions between compartments are provided in figure 1. There was no data on deaths due to undetected symptomatic cases and so we omit the corresponding transition.

There are two key differences in our model as compared to standard models. To model behaviour of the susceptible population, we adopt the modification of the transmission rate constant, β, used in standard models, by a function presented in [18] where the virus transmission rate is chosen to be dependent on the cumulative number of cases, decreasing as cases increase. Additionally, we have a population that was on lockdown (termed above as H). A release from lockdown transfers population from the corresponding lockdown compartments to the susceptible population, while lockdown policies transfer susceptible back to the lockdown compartments. This model has also been utilized in [18]. A related model, where lockdown policy impact was considered, has also been used in [13] where the lockdown was assumed to reduce the transmission by 90% but applied to the entire population. In our model design, this distinct compartment was considered isolated as we already use dynamic transmission rate to account for cautionary behaviour. This model limitation could be improved by considering transitions from this set of population to the exposed or infected sets.

### 2.1. Model usage overview

We used this model for projections of infection cases during the second half of 2021 (after June 2021) and early 2022. In India, NPI restrictions were removed starting in late January 2021 and lockdown re-introduced, during the second wave in April and May 2021, as illustrated by policy stringency levels [38] (detailed in §3.2.1). We used the increase in cases leading to the second wave in 2021 to quantify the growth of the susceptible population by estimating the rate of transfer of population from the lockdown population H into the susceptible population sets S. Together, this growth estimate and the estimated initial susceptible population formed the total susceptible population during the period from February 2021 to May 2021, and this estimate provided projections when policy restrictions were relaxed after June 2021. This baseline volume of population was used to determine the impact of different rates of release of population from H to S, the rate determined by the time period over which the transfer occurred. Typical time periods were 30 and 60 days, the two representing fast and moderate rates, respectively, of possible relaxation of government policies and social distancing behaviour. A linear release rate over the time period was assumed rather than an instantaneous transfer of population.

### 2.2. The compartment model

The change in population within compartments are represented by the following differential equations:


$$\begin{array}{rl} \frac{dH_{i}}{dt} & =v_{i}H_{i-1}-v_{i+1}H_{i}-\kappa_{i}(t)\;,\;i=0\ldots 4;\\ \frac{dS_{i}}{dt} & =v_{i}S_{i-1}-v_{i+1}S_{i}+\kappa_{i}(t)-\sum_{C}\frac{\beta_{i}^{C}S_{i}{\hat{I}}^{C}}{N}\;,\;i=0\ldots 4; \end{array}$$

where $\kappa_{i}(t)=\kappa (t)\cdot \frac{H_{i}}{\hat{H}}$ with κ(t) a function that is detailed later. This choice of $\kappa_{i}(t)$ represents an assumption of a homogeneous release, i.e., the release is not a function of the vaccination status since we have no information about the relative rates of release with respect to vaccination status. No bounds on the transfer of population from $H_{i}$ to $S_{i}$ were imposed. Furthermore, N is the size of the population, ${\hat{I}}^{C}=I^{C}+A^{C}$ and $\hat{H}=H_{0}+H_{1}+H_{2}+H_{3}+H_{4}$. We assume $v_{0}=0,v_{5}=0$. The transitions $v_{1},v_{2},v_{3},v_{4}$ represent transition rates between the population groups as represented in figure 1 and are obtained from the speed of vaccinations. And for each strain, C, of the virus the following differential equations govern, where we omit the notation for strain C for ease of understanding:


$$\begin{array}{rlrl} \frac{dE}{dt} & =\frac{\bar{\beta S}\cdot \hat{I}}{N}-\gamma_{E}E; & \frac{dI}{dt} & =(1-\alpha )\gamma_{E}E-(\gamma +\gamma_{2})I;\\ \frac{dA}{dt} & =\alpha \gamma_{E}E-\gamma_{3}A; & \frac{dI_{H}}{dt} & =\gamma I-(a_{1}+a_{2})I_{H};\\ \frac{dI_{N}}{dt} & =\gamma_{2}I-a_{3}I_{N}; & \frac{dD}{dt} & =a_{2}I_{H};\\ \frac{dR}{dt} & =a_{1}I_{H}+a_{3}I_{N}; \end{array}$$

where βS=β0S0+β1S1+β2S2+β3S3+β4S4. The model parameters, $\gamma_{E},\gamma ,\gamma_{2},\alpha ,a_{1},a_{2},a_{3}$ are all with respect to the strain C.

Each $\beta_{i}$ corresponds to the transmission rate of the virus in different vaccination status.


$$\begin{array}{rlr} & \beta_{1}=(1-\zeta_{1})\beta_{0},\; & \beta_{2}=(1-\zeta_{2})\beta_{0},\\ & \beta_{3}=(1-\zeta_{3})\beta_{0},\; & \beta_{4}=(1-\zeta_{4})\beta_{0}, \end{array}$$

and similarly, $\beta_{i}^{0}=(1-\zeta_{i}^{0})\beta_{0}^{0},i=1\ldots 4$ with $\zeta_{i}^{0}=\omega \zeta_{i}$, where ω is the loss of efficacy of the vaccine against Omicron variant and will be chosen from the set {0.5,0.25}. The value of $\zeta_{1}=0$ while $\zeta_{2}$ and $\zeta_{3}$ were chosen to be 0.65 and 0.8, respectively (V. K. Paul 2021, personal communication) and $\zeta_{4}=0.4$ as discussed earlier.

To model the impact of the virus on the population’s behaviour, we utilize a dynamic transmission rate utilized in [18], which leads to the value of $\beta_{0}$ as follows (for further discussion see [18]):


(2.1) $$\begin{array}{lcr} & \beta_{0}=\hat{\beta }\frac{1-c_{1}}{1-c_{1}\cdot \frac{S}{S_{I}}}=\hat{\beta }\frac{1-c_{1}}{1-c_{1}\cdot \frac{\left(S_{I}-G\right)}{S_{I}}} & \end{array}$$

where S is the total number of the current susceptible population, G the total number of reported cases, $S_{I}$ the total susceptible population (including the population estimated to be released) initially and $\hat{\beta }$ the initial transmission parameter at the start of the modelling. An illustration of how the transmission rate $\beta_{0}$ decreases with rise in infections is provided in the supplementary material (S2). In a more general approach to making $\beta_{0}$ dependent on the more recent infection can be modelled as


(2.2) $$\begin{array}{lcr} & \beta_{0}=\hat{\beta }\frac{1-c_{1}}{1-c_{1}\cdot \frac{S_{I}-wG}{S_{I}}}, & \end{array}$$

where wG is a weighted sum of reported cases, emphasizing the latest reported. The weighting for each reported case at day t,t≤T, is $r^{T-t}$, where T is the last day in the period under consideration and r is a factor that is estimated in the model.

The function κ(t) (again, note that $\kappa_{i}(t)$ is assumed independent of the vaccination status as mentioned earlier) is dependent on whether the policies are such that the population is in a release phase or in a lockdown phase. We use a linear function in our model:

— Release phase: In the release phase, between time $t_{1}$ and $t_{2}$, the function $\kappa (t)=h^{R},t_{1}\leq t\leq t_{2}$ where $h^{R}$ is a positive constant over the range of time $\left[t_{1},t_{2}\right]$, during which the population enters into a susceptible state. The value of $h^{R}$ is to be determined by the release policies and adherence.
— Lockdown phase: In the lockdown phase, between time $t_{3}$ and $t_{4}$, the function $\kappa (t)=h^{L},t_{3}\leq t\leq t_{4}$ where $h^{L}$ a negative constant, represents the rate at which the population withdraws into a state of lockdown, over the range of time $\left[t_{3},t_{4}\right]$. The value of $h^{L}$ is to be determined by the strength of the lockdown and adherence.

The theoretical value of the reproduction number, $R_{0}$, as analysed in an SEIR model with multiple virus compartments is provided in the electronic supplementary material. The analysis indicates that the variant with the highest transmission rate tends to dominate in the infection spread, which was also evident in our projections (see §4.2).

**Figure 2.**
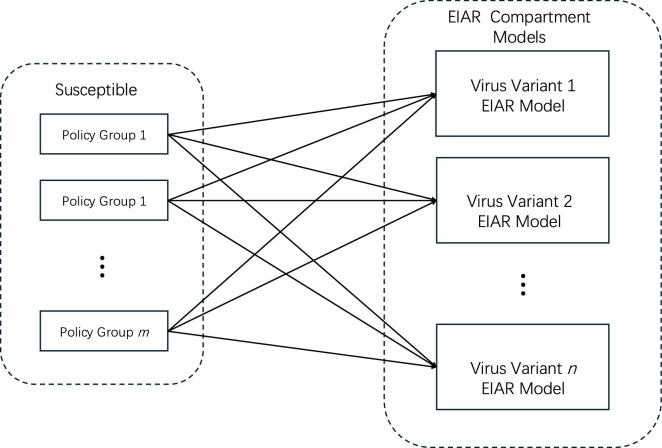
General model: Susceptible population is partitioned into Policy groups. Movement between policy groups is allowed. Infections will transition the population into the virus variant models (EIAR) where each policy group has EIAR transition model included for each policy group.

The above approach can be used in general situations where there are multiple policies and dynamic interchange of population following the policies (figure 2). One may have voluntary uptake of policies, e.g. mask following,vaccination, work-from-home, etc. These can be modelled by partitioning the susceptible set into subsets of population illustrated in figure 2.

Upon infection, the population transitions into a group that comprises compartments *E*, *I*, *A*, *R* as described above. We let $S_{i}$ be the susceptible in policy compartment i, and $A_{i}^{k},I_{i}^{k}$ be the undetected asymptomatic and infectious population following policy i and infected with virus variant k. Then susceptibles are infected at the following rate:


$$\frac{dS_{i}}{dt}=\sum_{j,k}\beta_{i,j,k}(I_{j}^{k}+A_{j}^{k})S_{i},$$

where $\beta_{i,j,k}$ is the transmission rate of the virus variant k from infected population following policy j onto population following policy i.

## 3. Model parameter estimation and projections

We detail here the methodology used for parameter estimation which will be used to obtain projections for both the Delta and the highly transmissible Omicron variant. We used a loss function along with constraints on the variables, specified by paramter ranges and minimized the error with respect to available data on confirmed cases and deaths. Penalty terms are incorporated into the loss function to determine solutions that lie in the interior of the feasible region defined by the parameter ranges.

Since the SEIAR-SD-L model was used to create projections in order to help guide policy advice for states in India, parameter estimation were based on individually fitting the model on each Indian state’s infection and death case data prior to the projection date. This captured the heterogeneity across states during the virus propagation. The results of the model for India were obtained by adding together the results estimated from each state’s model. The time span of the modelling was from 1 February 2021 until 30 November 2021, with projections reported after June 2021. For the Omicron phase, we estimated the transmissibility of Omicron variant using data from South Africa, where the Omicron wave was first reported, and compared the transmission rate with the previous variant’s transmission rate to compute a scaling factor that was applied to determine the transmission rate $\beta^{O}$ for the Omicron variant in India. The fitting for South Africa is detailed in the electronic supplementary material (electronic supplementary material, figure S4). Data for India and South Africa were obtained from [39,40]. In the absence of data on incubation period and infectious period for the Omicron variant in the beginning of December, we utilized the parameters of the Delta variant as estimated from our model during the phase where the Delta variant was prevalent. The vaccines were considered less effective with respect to the Omicron variant (as reported in December 2021 [41]). Due to lack of verified data on the efficacy reduction of the vaccines against the Omicron variant, we presented projections that are parameterized on the vaccine efficacy. These are detailed later.

### 3.1. Parameter estimation, baseline behaviour and assumptions

The baseline behaviour of the population was established between 1 February 2021 to 10 June 2021. Confirmed cases and deaths were used to estimate (i) the initial number of susceptible population, $S_{0}^{I}$ on 1 February 2021 and (ii) a release of population from lockdown into the existing susceptible pool during March 2021. The estimate of the release, modelled by the linear function $\kappa (t)=h_{1}$, along with the time span of release, $t_{1}$ to $t_{2}$, is established during model fitting in the first phase and is critical to establish a rise in cases. The time frame was chosen to span the second wave of infections. We incorporated the impact of reinfection by not limiting the total amount of population added into the susceptible compartment. Consideration of a SEIRS model that evaluates the impact of the virus on recovered population who are less susceptible due to antibodies, is left for further research.

The parameters established during model fitting in the first phase were used, after June 2021, for projections starting from the chosen start date T. Lockdown removal after June 2021 till the date T is also considered to account for release of population from the lockdown compartments to the susceptible sets. The parameters of this release, termed the second release, were estimated within a time period spanning from 18 June (after the peak of daily cases and when relaxation of policies was being considered; V. K. Paul 2021, personal communication) to the start date T of the projection period. This second release of population into the susceptible population is estimated via a second phase fitting procedure, keeping the parameters determined in the first phase as fixed except for the function κ(t). All the model parameters listed in electronic supplementary material, table S5, are determined individually for each state, while the assumed parameters are listed in table 2.

**Table 2.** Fixed parameters of the SEIAR-SD-L model for India. Data Source: [37] and

*v*_1_	— vaccination rate: 0.0015 (1 February 2021) 0.00225 (15 June 2021) 0.0036 (16 August 2021)	ζ_1_	0
*v*_2_	1/14 (2 week interval after first dose)	ζ_2_	0.65 (65% vaccine efficacy after first dose)
*v*_3_	1/84 (second dose interval)	ζ_3_	0.8 (80% vaccine efficacy after second dose)
*v*_4_	1/120 (4 month interval)	ζ_4_	0.4 (50% vaccine efficacy 4 months after second dose)

Model fitting parameters were determined by an optimization method to minimize a loss function that is dependent on case numbers and deaths. Limitations of a deterministic fitting [42], especially from the viewpoint of predicting uncertainty, will be discussed in the section on model limitations 5.1.

We let G,D denote the simulated cumulative cases and deaths, G,D are the corresponding reported data. Our optimization methodology utilizes random initial choices of the model parameters over defined ranges, as well as a search over the space of dates for population release from lockdown, to achieve the best fit. Ranges for these parameters are provided to the optimizer that minimized a loss function. Details of these ranges are provided in electronic supplementary material, table S5. Data for determining the ranges was established after considering values of the parameters available in the literature [43–48]. The range chosen for the incubation period, $\gamma_{E}$, was (0.15,4) [49] and for $\gamma ,\gamma_{2},\gamma_{3}$, the range was (0.08,0.4). The value of β was chosen in a range between 0.1 and 30 which encompassed reported contact paterns [50,51]. The optimization method used is L-BGFS that converges to a best-fit estimate of the model parameters, which due to the non-convex nature of the problem could possibly be a local optimum. Sampling multiple starting points to find the best fit leads to reasonably accurate fitting as measured by $R^{2}$ values.

#### 3.1.1. First-phase fitting

In the first phase, we determined the parameters related to the Delta variant of the virus. We consider the period from 1 February to 10 June 2021, as an effective first phase, which starts with a susceptible population, $S_{0}^{I}=\eta N$, where η<1 is a constant and N is the population. Additionally population is released into the susceptible population during a time period that is estimated via model fitting as we detail next. The release of the population from lockdown was assumed to be a linear function, with rate $\kappa (t)=h_{1}$ with a starting date $t_{1}$ chosen from the range of dates between 15 and 23 March. This range was a model choice based on the rapid rise in cases in March.

The other parameters of the linear release function are $h_{1}$ and the total size of the release, termed $\rho_{1}$; from which the ending date of the release $t_{2}$ can be deduced.

The parameters that are fitted in the first phase are $\beta ,\gamma_{E},\eta ,\alpha ,\gamma ,\gamma_{2},\gamma_{3},a_{1},a_{2},a_{3},h_{1},t_{1},\rho_{1}$. These parameters are used to generate the series G and D according to the differential equations governing the model. Ranges for these parameters are provided to the optimizer that minimized a loss function. Details of these ranges are provided in electronic supplementary material, table S5. These parameters were chosen to result in the best fit as determined by the optimizer.

The following loss function was used in the optimization:


$$\begin{array}{rl} & L_{1}(G,D,r_{ab})=\\ & -[\theta_{1}R^{2}(G,\bar{G})+\theta_{2}R^{2}(dG,d\bar{G})+\theta_{3}R^{2}(D,\bar{D})+\theta_{4}R^{2}(dD,d\bar{D})+\theta_{5}(1-(\frac{r_{ab}-\bar{r_{ab}}}{\bar{r_{ab}}}{)}^{2})]+\Delta_{1}, \end{array}$$

where the series G,D and G,D are weighted, emphasizing the latest data points in each phase. For the initial and reopen period of each series, the weighting for each entry at day $t,t\leq T_{1}$ is $0.98^{T_{1}-t}$, where $T_{1}$ is the end of the first phase. $R^{2}$ is the coefficient of determination, rab,rab are the antibody ratio at the end of the first phase of the simulation and the reported antibody ratio, respectively. In our model, $r_{a,b}$ is specified as:


$$r_{a,b}=\frac{R(T_{1})+S_{2}(T_{1})+S_{3}(T_{1})+H_{2}(T_{1})+H_{3}(T_{1})}{S(0)+H(0)},$$

where S(0) and H(0) are the total susceptible and lockdown population at the beginning of the time period (1 February 2021). We assumed no loss of vaccine efficacy during this initial period. We obtained rab from sero-surveys carried out by ICMR [52–54] and applied an average value (40%) for the first phase (details may be found in the code). $\Delta_{1}$ is a penalty function that penalizes parameter choices that come close to the boundary of the parameter’s range. It is the average error from the following parameters : $\beta ,\frac{1}{\gamma_{E}},\eta ,\frac{1}{\gamma },\frac{1}{\gamma_{2}},\frac{1}{\gamma_{3}},\frac{\rho_{1}}{\eta N}$. For each parameter, para with lower and upper bound L,U, its error is


$$\frac{\max(|para-\frac{L+U}{2}|-0.35,0)}{U-L}.$$

Our simulation is on a daily basis with $dG,dD,d\bar{G},d\bar{D}$ being calculated daily. After experiments, we chose the parameters in the loss function as $\theta_{1}=\theta_{2}=0.35$ and $\theta_{3}=\theta_{4}=(0.9-\theta_{1})/2=0.1$ and $\theta_{5}=0.1$ emphasizing infection cases over deaths since our goal was projections of confirmed cases.

#### 3.1.2. Second-phase fitting for reopen

In the second phase, we considered the period from 10 June 2021 till the end date of fitting, T, which was also the start of future projections. This spanned the period within which the release of people from lockdown, after the second wave, occurred. All parameters determined in the first phase were fixed for the second phase. The release function κ(t) was estimated as a linear function with parameters as discussed in the model. We estimate the rate of the second release, termed $h_{2}=h^{R}$, the start date of release and the total size of the release, termed $\rho_{2}$. The starting date was within the range from 18 June 2021 till two weeks before the end of the fitting period. The ending date was determined by $h_{2}$ and the size of the release.

The estimation again uses an optimization method with the following loss function:


$$L_{2}(h_{R})=-[\theta_{1}R^{2}(G,\bar{G})+\theta_{2}R^{2}(dG,d\bar{G})+\theta_{3}R^{2}(D,\bar{D})+\theta_{4}R^{2}(dD,d\bar{D})]+\Delta_{2},$$

where we chose $\theta_{1}=\theta_{2}=0.40$, $\theta_{3}=0.1$, $\theta_{4}=0.1$. Antibody ratios were not used as we had no sero-survey data during this period and re-infections were possible. The series G,D and G,D contain dates only in the second phase and are weighted in the same way as in the first phase. $\Delta_{2}$ is a penalty function similar to $\Delta_{1}$ applied to the parameter $h_{2}$.

#### 3.1.3. Estimated model parameter discussion

We tested the fitting with the two dynamic β functions that were discussed in the model section. Our model fitting had very high $R^{2}(0.998)$ for confirmed cases using the dynamic beta function in equation (2.1). Given the number of parameters in the model, it is likely that multiple solutions would result in very good fitting. The parameters determined when the fitting was done with the first β function defined by equation (2.1) is reported here. The second dynamic β defined in equation (2.2) function resulted in parameters that had far more variations and needs further study. For the model with the dynamic β function in equation (2.1) we determined that:

— β: had a range of (4.42,8.36) with a mean of 4.98.
— $\gamma_{E}:$ had a range of (0.17,0.32) corresponding to range of 3.1–6 days (mean 4.5 days) of incubation days of the virus.
— γ: had a range of (0.09,0.25) with a mean of 0.19 corresponding to 4–11 days (mean 5.4 days) of infectious period before hospitalization.
— $\gamma_{2}:$ the infectious period for symptomatic population who recovered without hospitalization had a range of (0.09,0.399) corresponding to an infectious period of 2.5–11 days (mean 4.8 days).
— $\gamma_{3}:$ the infectious period for asymptomatic population had a range of (0.09,0.25) corresponding to infectious period of 4–11 days (mean 4.7 days).
— The fraction of the population that was asymptomatic had a range of (26, 90)% and a mean of 78%.

#### 3.1.4. Testing the parameter fitting accuracy

##### 3.1.4.1. Accuracy of fitting phase up to May

We also determined the accuracy of our fitting. To do so we estimated parameters for our model using data until 11 May 2021. We fit parameters for the first phase of our model using four sets of experiments where we used training data until 11 April 2021, 18 April 2021, 24 April 2021, 30 April 2021 and estimated the error in projections of daily cases of infections and deaths at dates that were 1−4 weeks beyond the fitting dates. The error in projections of daily cases beyond the four chosen training dates was between 8.4% and 14.2% over a one week period as detailed in the supplementary material (electronic supplementary material, tables S12 in section S3). Since the vaccination rates were still low, the projections used for testing the accuracy of the model did not account for vaccinations.

##### 3.1.4.2. Accuracy of fitting phase from June onwards

**Figure 3.**
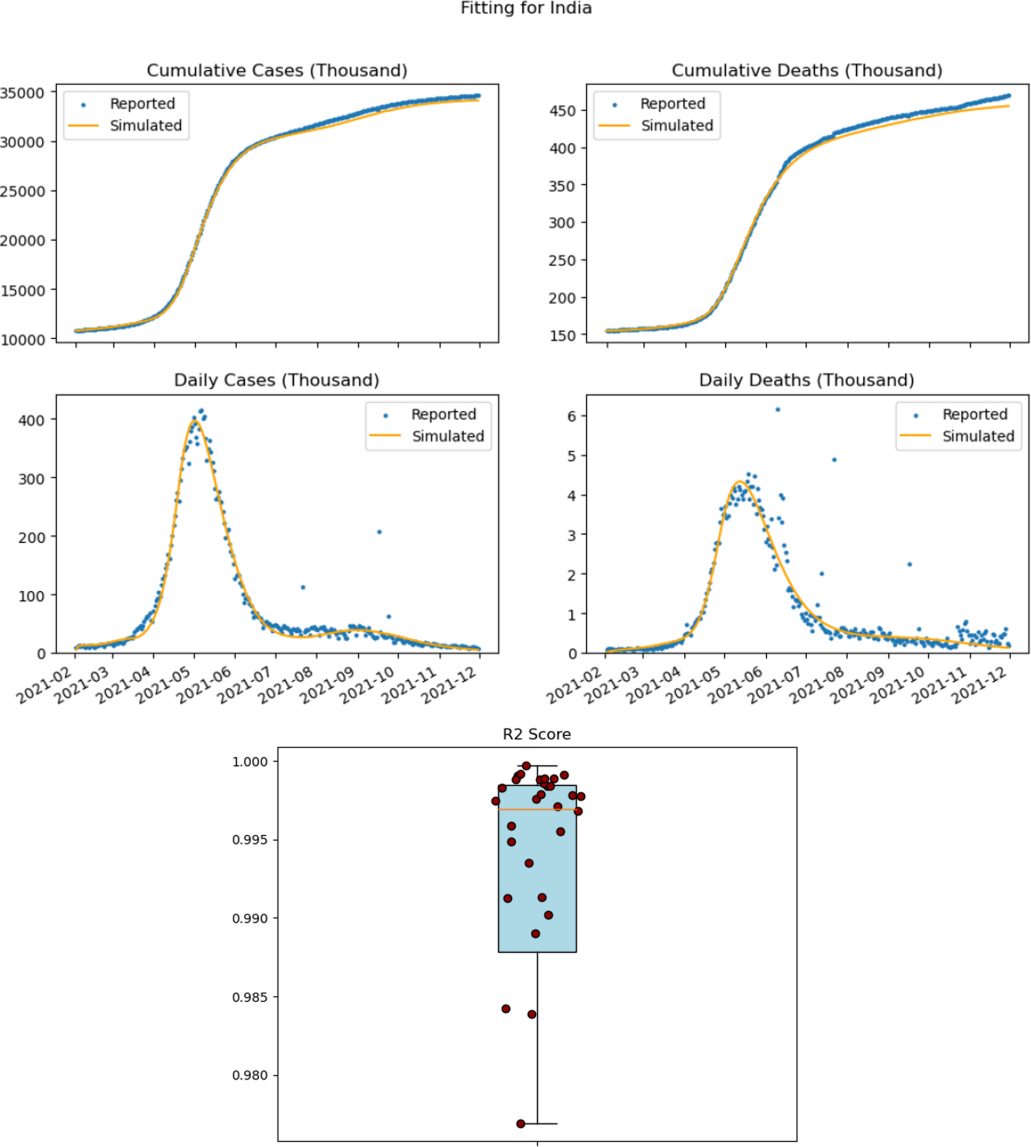
Fitting results for India, obtained by the sum of the fitting data for each state within India, illustrating model performance for both confirmed cases and deaths during the period from 1 February to 15 December 2021. Actual data indicating cumulative numbers for each state was used for the fitting and the model's performanceas measured by$R^{2}$ scores for each of the states illustrates the accuracy of the model. Outliers were removed from the box plot. Outlier data are in the electronic supplementary material, figure S2

We illustrate our fitting for the combined first and second phase in figure 3. $R^{2}$ values illustrating the goodness of fit was determined and listed in electronic supplementary material, table S4. The box plot in figure 3 illustrates that the range of $R^{2}$ scores is close to 1.

### 3.2. Model-based historical projections

**Figure 4.**
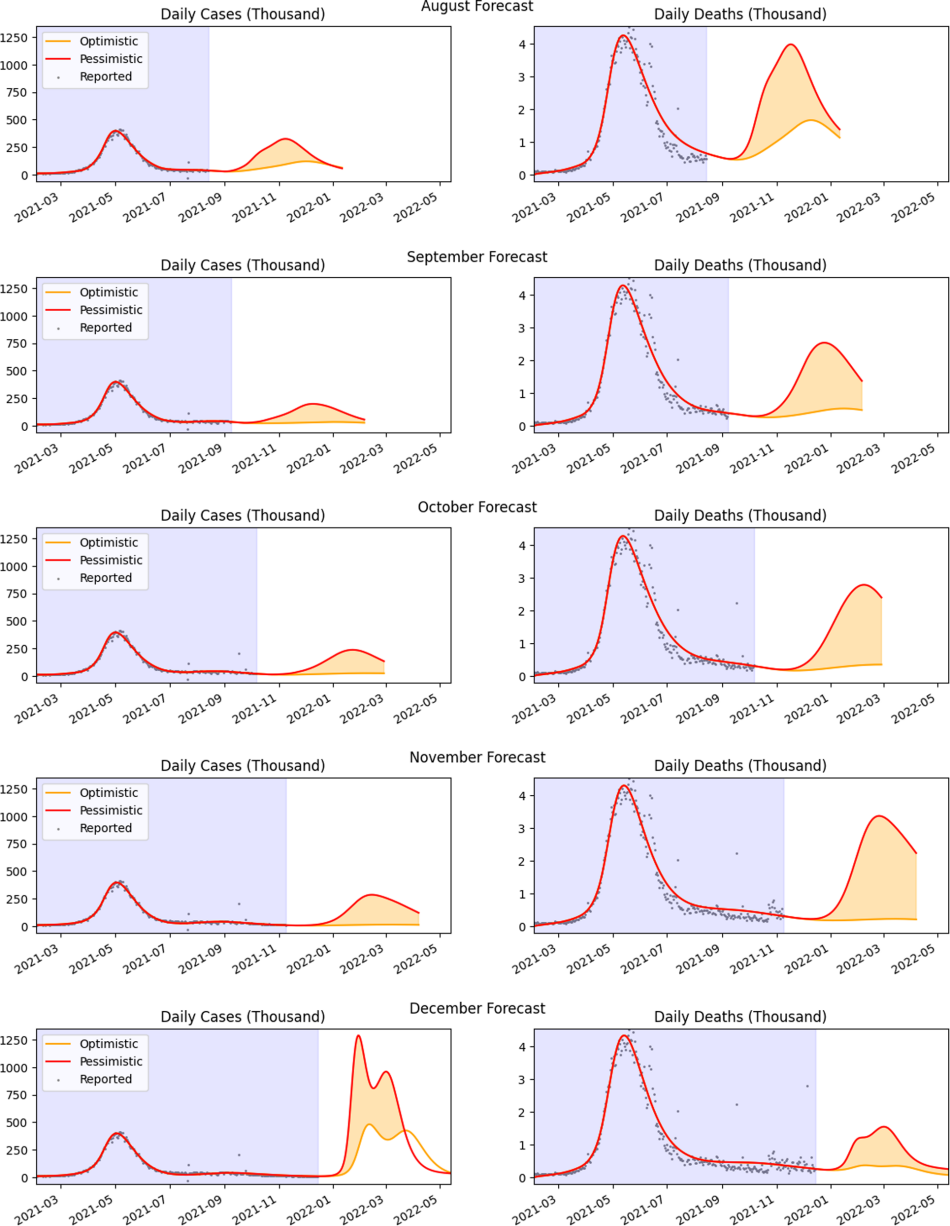
Monthly sequence of projections for the case of fast release rate. The peak case load varied according to the current infection trends and successively delayed by almost a month. Model fitting was conducted using data from the shaded period.

In this section, we present results based on reports that we had periodically submitted to NITI Aayog on projections for confirmed cases and deaths when lockdown policies were relaxed during the months from June to December 2021 (figure 4 and table 3). The model parameters used were different from the ones presented in §3.1.

**Table 3.** Summary of model-based recommendations.

report date	report summary
9 June 2021	Doubling of vaccination from current 2 million per day will reduce cases and deaths by 14% and 12%, respectively. Slow relaxation of restrictive policies over 6 months will reduce cases and deaths by 40% and 32%, respectively. This is in comparison to relaxation of policies over 30 days. Pessimistic scenario 300 000 peak cases with vaccines. Only pessimistic scenario estimated.
26 July 2021	Vaccination rates increased to 3 million per day. Relaxation of restrictive policies over 2 months will reduce risk of third wave and provide substantial benefits to cases (80% and 76% for cases and deaths), as compared to relaxation over 1 month. Only pessimistic scenario predicting 160 000 peak cases estimated.
19 August 2021	Vaccination rates 3 million per day. Stagnation in number of cases noted. Slower relaxation of restrictions will reduce pessimistic projections of cases and deaths by 69% and 62%, respectively. States with higher growth patterns include: Andhra Pradesh, Delhi, Gujarat, Himachal Pradesh, Karnataka, Maharashtra, Kerala, Punjab, Rajasthan, Uttarakhand and some NE states. Optimistic scenario showing steady decrease in cases (range of 325 000−120 000 peak cases within 1−15 November).
12 September 2021	Vaccination rates have increased to 5 M/day. Reduced rate of removal of restrictive policies will eliminate any further peaks. Slow relaxation over 60 days will reduce cases and deaths even further (81% and 79%). Some signs of growth of infections appear in Chandigarh, Delhi, Gujarat, Himachal Pradesh, Karnataka, Maharashtra, Madhya Pradesh, Punjab, Tamil Nadu, Uttar Pradesh and Uttarakhand (range of 198 000−35 000 peak cases within 1−15 December).
12 October 2021	Good progress over last 4 months, however infection cases persist due to reduction in stringency measures. With an increased pace of relaxation of norms increased cases are anticipated in November, December and January. Peaks can be eliminated via strict social distancing measures (range of 237 000−25 000 peak cases within 15−30 January).
16 November 2021	The likelihood of further peaks of infections has been delayed to January 2022. Majority infections will be for nonvaccinated populations or incomplete vaccinations (approx. 75%). Risks will increase with removal of restrictive policies (range of 285 000−14 000 peak cases within 15−28 February).
11 December 2021	Projections include Omicron variant (vaccine efficacy against Omicron estimated to be 50%). Substantial rise in cases are projected in January with peak cases varying between 600 000 and 2 000 000 in the optimistic and pessimistic scenarios. Implementation of substantial restrictions will further reduce the peak cases to a range of 300 000–700 000 (range of 2 000 000−600 000 peak cases within 1−15 February).
8 January 2022	Projections including Omicron, with vaccine efficacy reducing over time. Number of cases in pessimistic scenario reduced to maximum peak of 1.25 million (corrected from initially estimated 1.6 million) cases. Mild lockdown policy implementation (20%) accounted for on 10 January 2022 with gradual release. Mild lockdown will reduce infection case range to between 600 000 and 150 000 (range of 1 290 000−480 000 within 1−15 February).

Our projections considered two scenarios based on the size of the future release of population, $\rho_{F}$, from the lockdown compartments $H_{i},i=0\ldots 4$ into the corresponding susceptible pool, $S_{i}$. In each scenario, the projections were started with the population size of the compartments as obtained from the model at the end of the fitting phase.

Scenario 1 (pessimistic scenario–red curve): The pessimistic scenario is when the number of population of the future release into susceptible pool, $\rho_{F}$ is equal to the release of population established during the baseline period, $\rho_{1}$, obtained from the estimated linear rate $h_{1}$, together with the estimated susceptible population, $S_{0}^{I}$, at the start of the baseline period, i.e. $\rho_{F}=S_{0}^{I}+\rho_{1}$. The release is chosen to reflect unrestricted population behaviour on removal of policy mandates, similar to that in the period 1 February 2021 to June 2021.

Scenario 2 (optimistic scenario–orange curve): This scenario assumes that the size of the second release after the relaxation of lockdown in June 2021 till the end of fitting, termed $\rho_{2}$, is discounted from the future release of population into the susceptible pool, i.e. $\rho_{F}=S_{0}^{I}+\rho_{1}-\rho_{2}$ (electronic supplementary material, table S6). This is an optimistic scenario which accounts for reduced susceptible size due to the relaxation of policies after June 2021 and spreading infection.

Projected release rate from lockdown: To account for the susceptible population increasing at a substantial rate upon relaxation of policy stringency, we considered two possible release rates of the population from the lockdown compartments into the susceptible sets, starting from a specified release date. The two release rates were obtained by computing the release of the estimated base population, $\rho_{F}$, in 30 and 60 days, termed as fast release and moderate release, respectively,

We illustrate the succession of projections for the fast release rate (30-day release) in figure 4 starting in the month of August 2021. From August 2021, our projections’ range is bounded by the two scenarios, pessimistic and optimistic, discussed above. For the projections in December, 2021, that included the Omicron variant, vaccine efficacy of 50% is assumed to compute the projections illustrated in figure 4. A summary of estimates provided in our reports is contained in table 3. The set of policy documents generated and submitted to NITI Aayog is provided in the electronic supplementary material, S6. The code used is available in the Zenodo public repository [55].

Over the period from June till December of 2021, our model projections showed a reduction in cases and then an increase during the winter months ending in a sharp rise in predicted cases when the Omicron variant started infections in the population and became prevalent. Our initial lower projections of the growth of infections during August, September and October could possibly be attributed to government actions including slow release from lockdown state and enhanced vaccination rates.

The results of the projections for Indian states for the third (Omicron) wave were indicated in a report in December, 2021 and updated in early January, 2022 (see table 3 and electronic supplementary material, S6). Our projections were based on initial number of 50 Omicron cases in the infectious compartment, $I^{O}$, within each state on 15 December 2021, the number chosen due to the absence of confirmed numbers (accurate Omicron variant case numbers were unknown).

For the case of the pessimistic scenario, the results indicated a substantial rise in infections when rules regarding lockdown are relaxed. Our results projected that there would be increasingly high daily case loads peaking around the end of January 2022 and the beginning of February 2022, extending into March and April months. Our model had indicated infection cases peaking at almost 1.3 million cases per day for an assumed 50% vaccine efficacy loss against the Omicron variant. This indicated that relaxation of policies should be reversed and utmost care be taken to ensure social distancing and mask usage, reduced capacity of interior locations and marketplaces, especially in restaurants and bars based on earlier research in [18]. As detailed earlier, we assume that in the optimistic scenario the population that is added to the susceptible set after relaxation of lockdown in June 2021, but before 15 December 2021, is discounted from future release of population into the susceptible pool that occurs after 16 December 2021 (electronic supplementary material, table S6). Even in this scenario, the projections indicated a substantial rise in daily infection case numbers peaking at above 400 thousand cases.

Our projections in January 2022 are higher than the reported confirmed cases. This reduction in the rise of reported cases was likely due to the spread of the virus being tempered by restrictive government policies, that included night curfews as well as business and school closings during January (an example is the policy in the state of Delhi [36]).

The dynamic progression of infection case projections is illustrated in the accompanying video [56].

#### 3.2.1. Estimating impact of model projections on policy stringency

**Figure 5.**
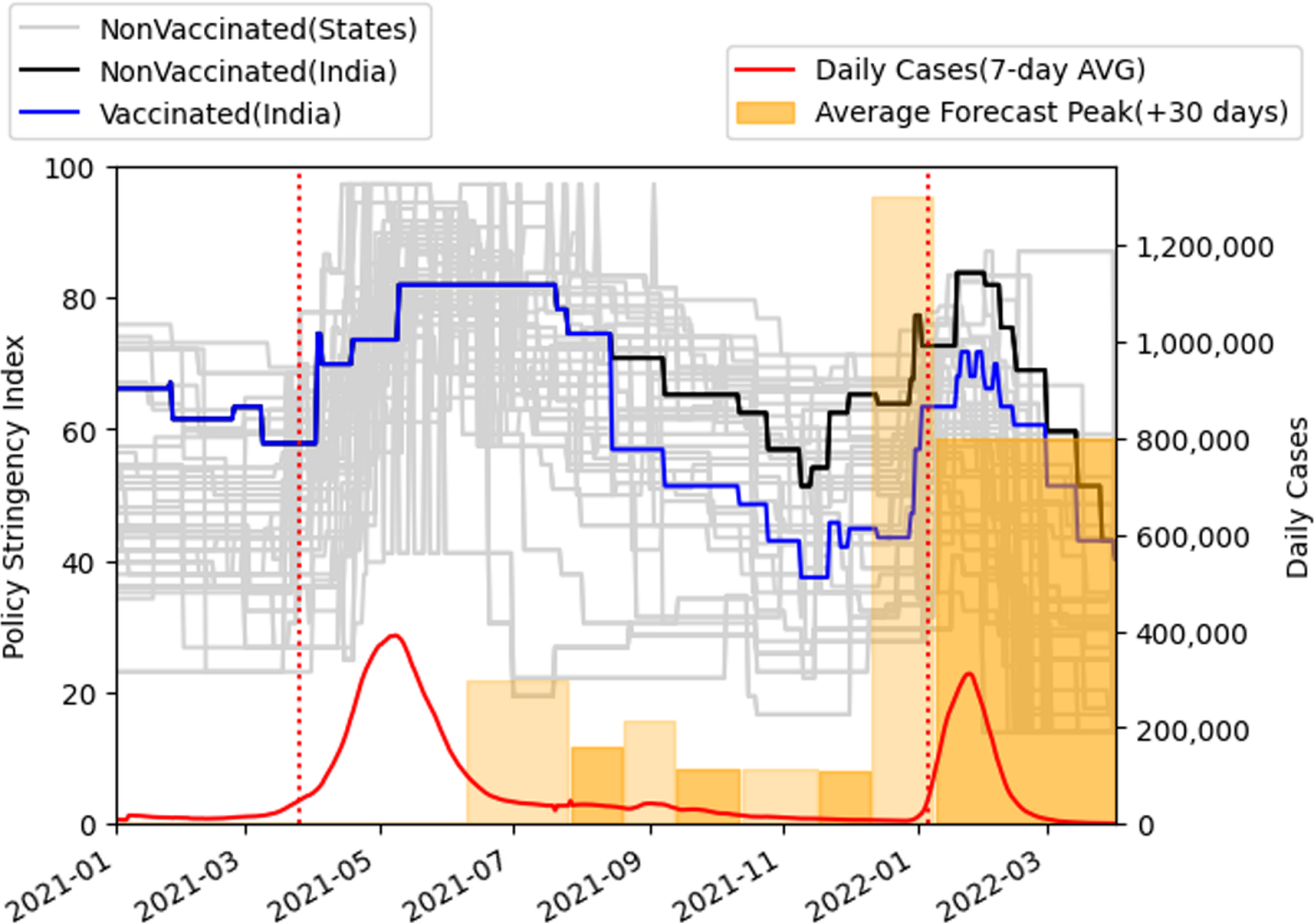
Causality between SIR-SD-L model predictions (average forecast peak as coloured bars over the time sequence) and Indian government policy stringency level (courtesy: Oxford Stringency Tracker [38]). The grey lines represent the Indian states stringency index. The 7-day average confirmed daily COVID-19 infection cases showed a lag as compared to the predictions. Stringency policy appeared preemptive.

Policy decisions during Covid-19 were complicated due to multiple disease factors, vaccination rates and economic factors, and were likely informed by multiple experts and models. While it is difficult to measure the impact that our sequence of projections had on policy, we present our projections in the context of the stringency of the policies implemented. We use data from the project on monitoring government policies at Oxford University [38]. This is illustrated in figure 5, which shows the stringency level of the national policies both for vaccinated and unvaccinated groups. Also illustrated is the timeline of our average peak forecasts (approximated to thousands) as summarized in table 3. The average is computed over the pessimistic and optimistic peak projections of our model as reported on the dates listed in table 3 for the case of fast release (30-day period). These successive forecasts are illustrated by the coloured bars along with the stringency index over the projection timeframe. To measure the impact of our projections on policies, we measured the difference between the stringency index at the beginning of the second and third waves of the COVID infections. As illustrated in the figure, the national policy stringency when the 7-day average of the daily infected cases exceeded 50 000 cases during the second phase was 57.87, whereas in the third phase, it was 72.69. We further investigated causality between our policy predictions and stringency levels. Results indicate that the average forecast peak value policy predicted (or Granger caused) the stringency index as indicated by the Granger-causality test. For unvaccinated stringency policies and a 24-day lag, the ssr-based *F* test results were: *F* = 1.9570, *p* = 0.0064 , d.f. (denom) = 223, d.f. (num) = 24, while for vaccinated stringency policies and a 24-day lag, the ssr-based *F* test results were: *F* = 1.7668 , *p* = 0.0180 , d.f. (denom) = 223, d.f. (num) = 24.

One may observe the rapid rise of projection of confirmed cases due to the Omicron variant.

## 4. Projections for the Omicron wave

In this section, we present projections based on our model for the Omicron wave. Our projections in this section are based on the updated parameters obtained from current range bounds.

### 4.1. Projection accuracy for the Omicron wave

**Figure 6.**
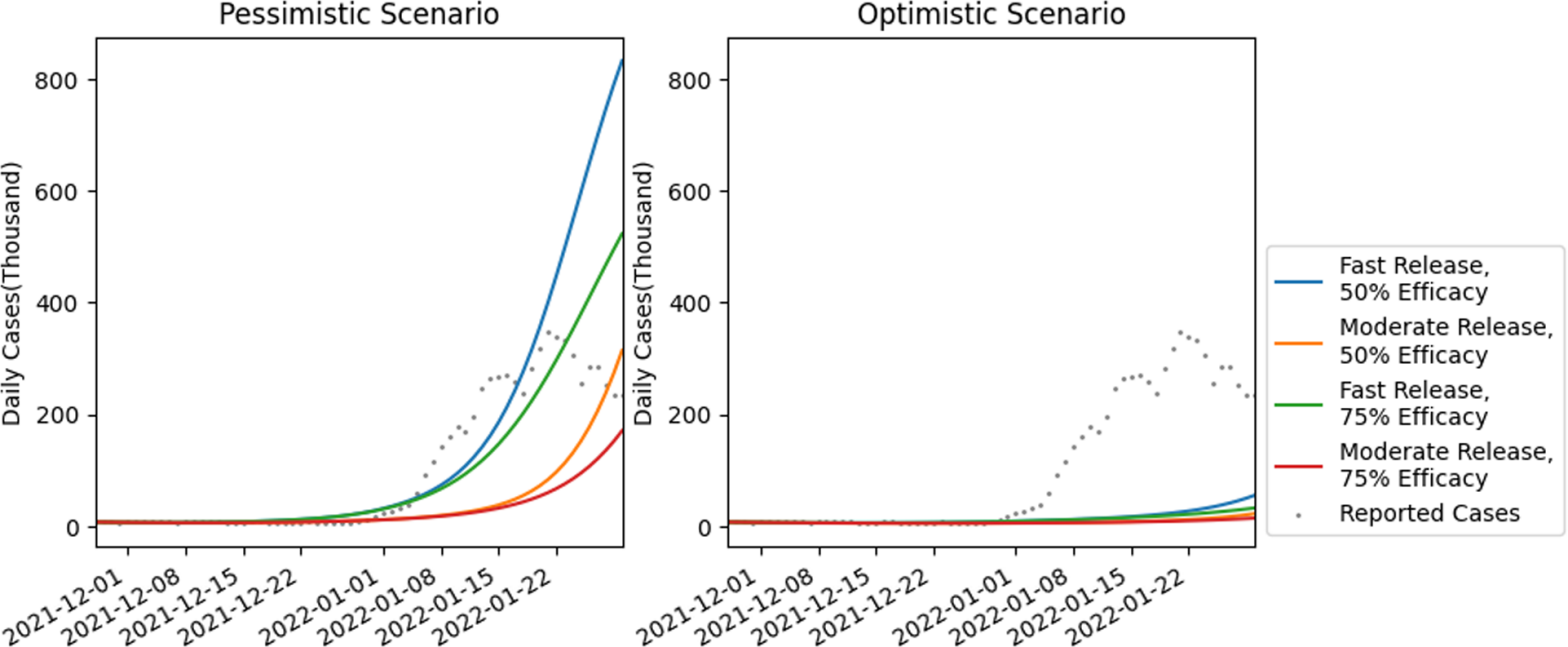
Daily infection case comparison between projections and reported cases in the pessimistic and optimistic scenarios during the Omicron wave. The projections started on 1 December.

The projection accuracy was measured by evaluating mean absolute percentage error (MAPE) values comparing the projections and the actual confirmed cases (electronic supplementary material, table S3). For the fast release (30 days), we compared the error in our pessimistic estimates versus the actual data for cumulative and daily confirmed cases and deaths at three time intervals, corresponding to 15, 30 and 45 days starting from 30 November 2021 (see also figure 6). Cumulative cases were counted from 30 November 2021. The MAPE measure for cumulative cases was 18.13%, 15.4% and 18.9% for 15-, 30- and 45-day intervals, respectively. The MAPE values for daily cases were 18.25%, 56.5% and 51.5% for the 15-, 30- and 45-day interval projections, respectively (electronic supplementary material, table S3). The death projections were worse. The accuracy measured above is possibly further impacted as it did not account for the NPI measures enacted subsequently in late December 2021 and January 2022 (an example of the restrictions in the state of Delhi can be found in [36]). The possible impact of NPI measures based on partial lockdown is presented in the next section.

### 4.2. Policy impact analysis: incorporating policies into model projections

**Figure 7.**
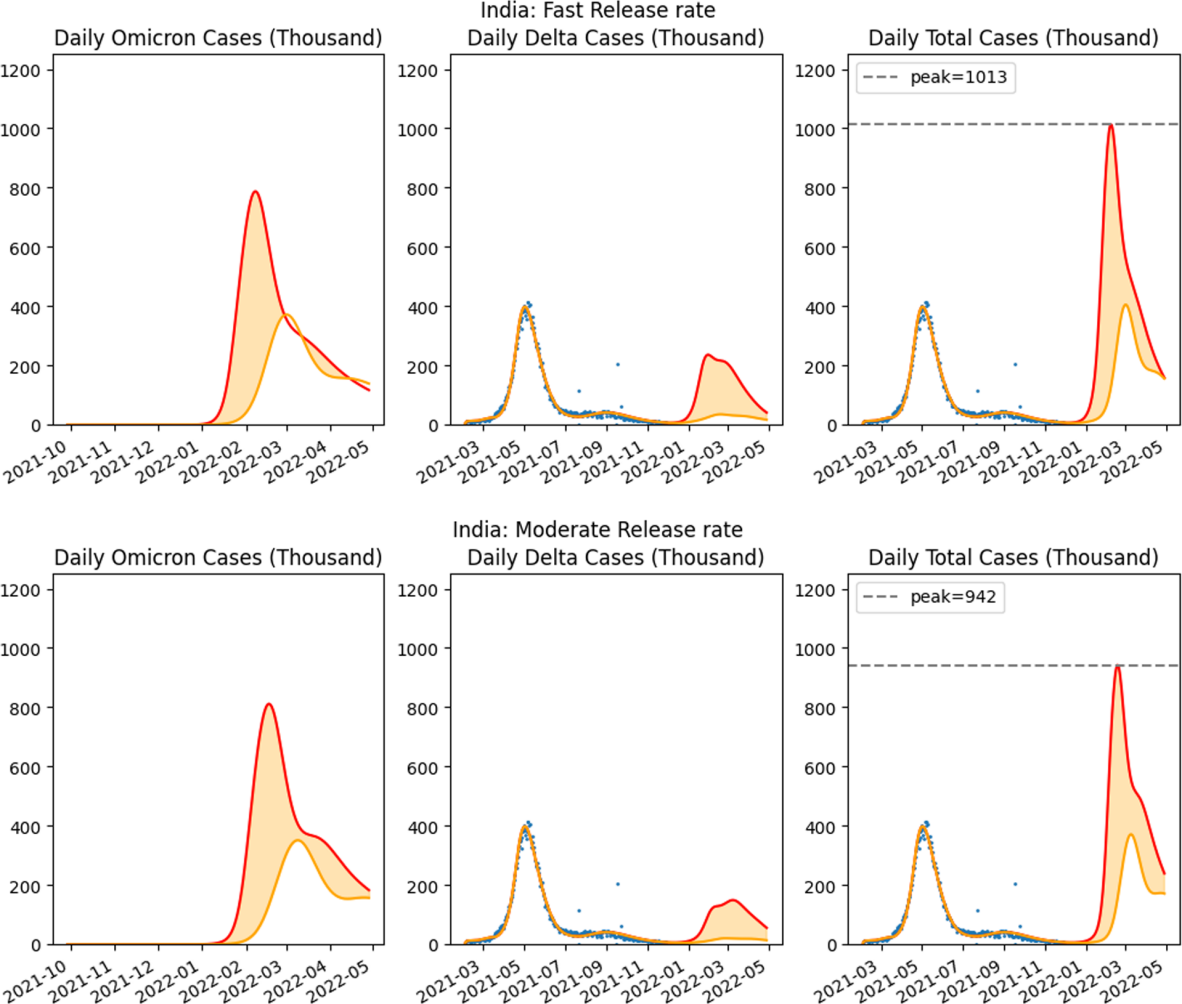
Fifty per cent vaccine efficacy against Omicron, No lockdown: Comparison of two scenarios: release of population from lockdown with two release rates, fast (30 days) and moderate (60 days). Red indicates the worst case behaviour (pessimistic scenario) and orange indicate the optimistic scenario. The leftmost graph in each row is for the projected cases of the Omicron variant, whereas the third graph is for the projected confirmed cases of the two variants together.

The projections of our modelling showed very high peaks in the confirmed cases for the Omicron phase (figure 7 and electronic supplementary material, figure S5). As detailed earlier, we modelled this release in two cases, where the susceptible population is increasing due to a fast release and a moderate release. The estimates illustrate mixed impact of the two variants, with the Omicron variant rapidly dominating the currently existing Delta variant.

Since one of the possible NPI policies was partial lockdown, we considered the impact of lockdown on the case numbers. We determined projection estimates from our parameterized model when there was no lockdown policy and compared these to the situation when there was partial lockdown initiated. In the discussion below, we provided details of the projections when there is partial lockdown that reduces the susceptible population by a factor of 15%, 20% and 25%. The cases, based on vaccine efficacy and lockdown assumptions and the figures corresponding to the projections are listed in table 4.

**Table 4.** Cases considered during the Omicron phase. Fast release corresponds to the release of population in 30 days and moderate release release over 60 days.

scenario	release type
vaccine efficacy 50% and no lockdown	fast and moderate release illustrated in figure 7
vaccine efficacy 75% and no lockdown	fast and moderate release illustrated in electronic supplementary material, figure S5
vaccine efficacy 50% and {15,20,25}% lockdown	fast release illustrated in figure 8
vaccine efficacy 75% and {15,20,25}% lockdown	fast release illustrated in electronic supplementary material, figure S6

**Figure 8.**
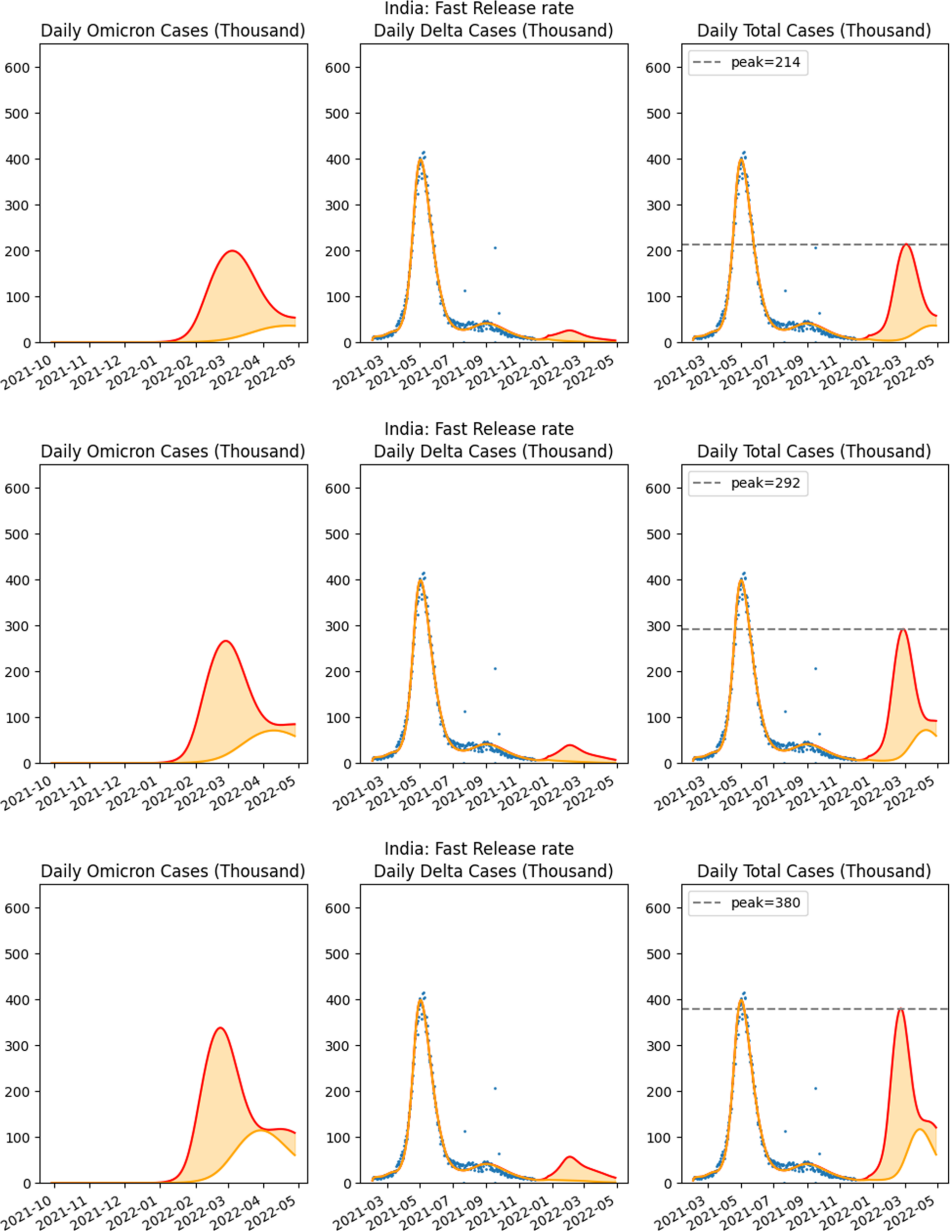
Fifty per cent vaccine efficacy against Omicron with lockdown proportion 25%, 20% and 15% starting 23 December. Comparison of three scenarios based on the proportion of population under lockdown. The leftmost graph in each row is for the projected cases of the Omicron variant, whereas the third graph is for the projected confirmed cases of the two variants together.

### 4.3. Impact of lockdown during Omicron wave

As detailed earlier and illustrated in figure 7 and electronic supplementary material, figure S5, the projections indicated a substantial rise in infections when rules regarding lockdown are relaxed. Note the pessimistic outcome in the fast release scenario has projected infection cases peaking at around 1.01 million cases per day for 50% vaccine efficacy and 0.75 million cases for 75% vaccine efficacy.

We also consider the situation when a mild lockdown is implemented after mid-December when substantial cases started being reported. December 23 was chosen as the lockdown date in our simulation. This lockdown is assumed to reduce the susceptible population by 15%, 20% and 25% for the fast release rate scenario (30-day release) prior to the date of lockdown. It is assumed that the release of population stops, and is actually reversed, as the lockdown is implemented. In the case of 50% vaccine efficacy, we observe benefits due to partial lockdown. With 25%, 20% and 15% lockdown, starting on 23 December 2022, the predicted pessimistic peak number of confirmed infected cases are 214 000, 292 000 and 380 000, respectively, as compared to the pessimistic prediction of 1 013 000 cases when no lockdown is enforced (figure 8), resulting in less cases and consequent hospitalization with less impact on the health system.

**Figure 9.**
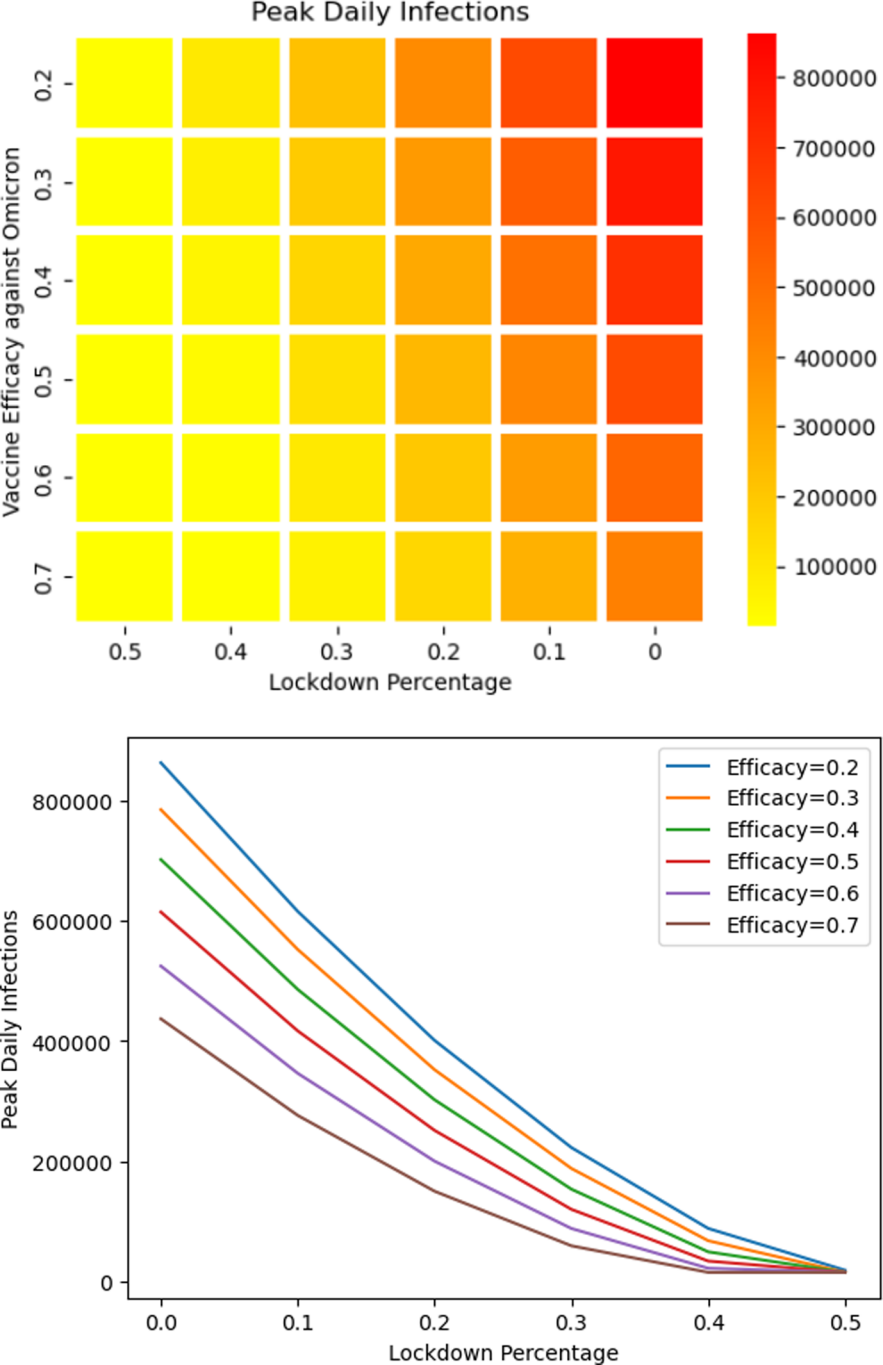
Predicted peak of confirmed cases as dependent on efficacy of vaccine against the Omicron variant and percentage of population under lockdown. The heat map shows the progression of decrease of cases with increased vaccine efficiency. The lockdown percentage has a more pronounced impact towards decreasing peak daily cases. These illustrations are for the fast release rate scenario. The release stops at the time of lockdown.

**Figure 10.**
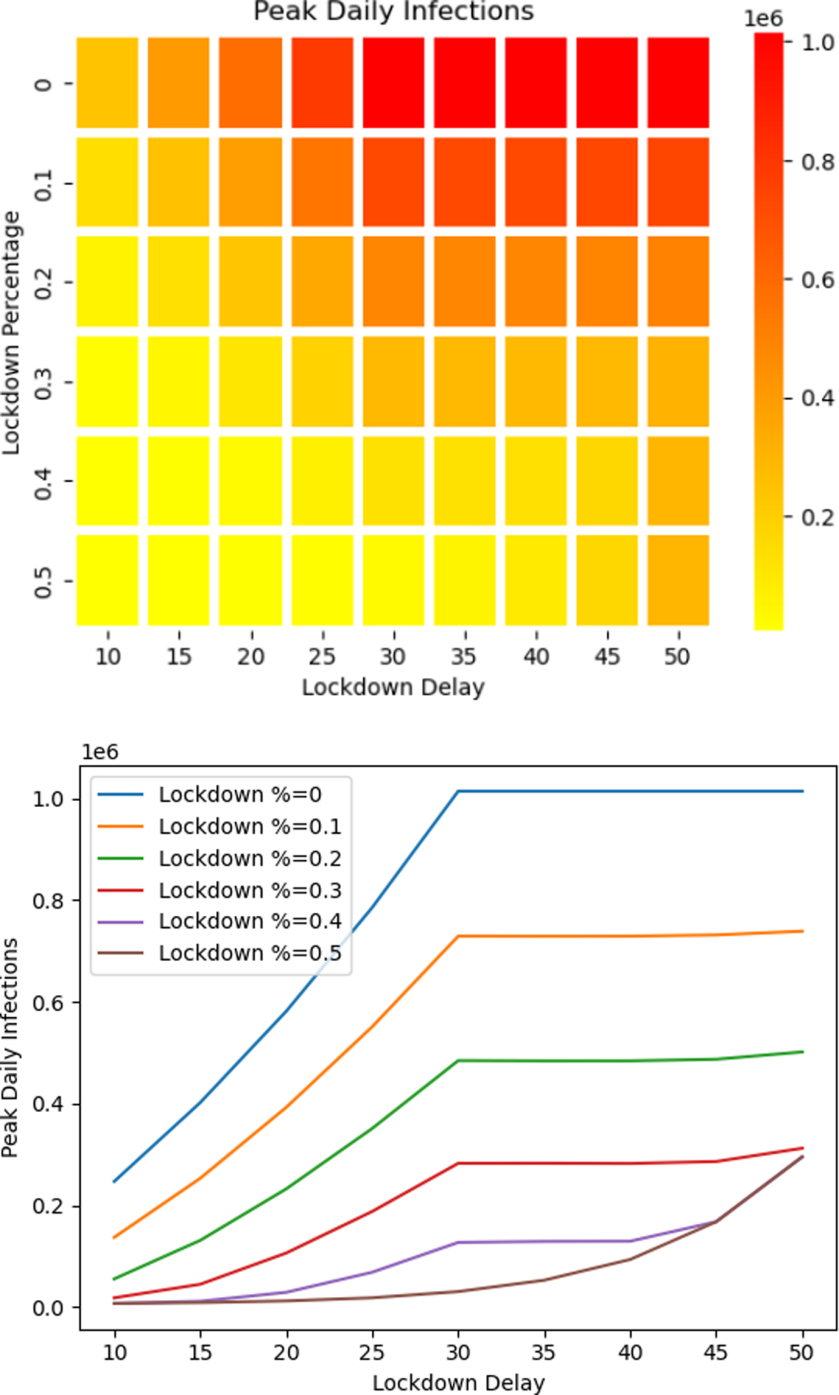
Predicted peak of confirmed cases as dependent on percentage of population under lockdown and delay of lockdown. These illustrations are for the fast release rate (30 days) scenario. The release stops at the time of lockdown. For policies that enforced low lockdown percentages, delaying the lockdown date beyond 30 days resulted in no or little change to the high number of daily peak cases.

At the end of November 2021, stringency levels were low (figure 5) [38], and the population was slowly resuming normal behaviour. At the onset of the Omicron variant, the benefits of partial lockdown were considered and illustrated in figure 9 where the peak number of confirmed cases shows an almost linear decrease with respect to the increase in the percentage of people in partial lockdown. Furthermore, figure 10 shows that if new population is added into the susceptible set quickly then delaying partial lockdown increases the number of peak daily infections, especially when the percentage of population under lockdown is low. There is no benefit to be gained from delaying lockdown beyond the date when new population is added.

## 5. Conclusions and limitations

Early warning systems which project infection cases growth more than a month in advance, while not able to pinpoint exact peaks and accurate case projections, are very useful for policy decisions. Our results were made available to NITI Aayog (a think tank at the central government in India) to help with policy decisions. Partial lockdowns, based on inputs from multiple sources, were implemented by various state governments in India.

As indicated from the results, which re-evaluated projections monthly, long-term projection of the disease is impacted by governmental policy decisions and adherence to social distancing policies by the population. It appears that policy decisions, including vaccinations, prevented a substantial rise in cases until the third wave in December 2021; however, further investigation of the impact of the policies is required. While we have accounted for measured relaxation of lockdown until December 2021 and investigated different levels of lockdown strategies in January 2022, the impact of a variety of factors including adherence to policy declarations remains an important aspect of further study [57].

### 5.1. Limitations of the model and method

A key feature of our model is the use of a compartment that added an extra state of the population in lockdown mode as opposed to incorporating lockdown effects modelled via a lowered disease transmission rate β. The functions that model β need to be further studied regarding the use of both short-term and long-term memory features. Furthermore, our model uses a linear rate of release of the susceptible population. Replacing this with other release functions could possibly result in improved accuracy.

Moreover, behaviour changes of vaccinated population also need to be addressed as vaccinated people may not follow strict social-distancing guidelines. The use of vaccination status in infectious and hospitalized compartments should also be considered. Additionally, other socio-economic factors may need to be incorporated into the model, as well as the possibility of re-infections of the population.

The pessimistic and optimistic scenarios reported could be considered in a more fine-grained approach and correlated with policies. Furthermore, the current opitmization approach for determining model parameters has limitations and other approaches could help in predicting uncertainties [42].

## Data Availability

Data and relevant code for this research work are stored in GitHub (https://github.com/sanjivk1/COVID-19-INDIA.git) and have been archived within the Zenodo repository [55]. Supplementary material is available online [58].
